# Promotion of Food and Beverages by German-Speaking Influencers Popular with Adolescents on TikTok, YouTube and Instagram

**DOI:** 10.3390/ijerph191710911

**Published:** 2022-09-01

**Authors:** Eva Winzer, Brigitte Naderer, Simeon Klein, Leah Lercher, Maria Wakolbinger

**Affiliations:** 1 Department of Social and Preventive Medicine, Center for Public Health, Medical University of Vienna, 1090 Vienna, Austria; 2 Department of Media & Communication, Ludwig-Maximilians University of Munich, 80539 Munich, Germany

**Keywords:** food cues, influencer food marketing, nutritionally poor food, adolescents

## Abstract

The promotion of nutritionally poor food and beverages (F&B) has a proven effect on children’s eating preferences and, therefore, plays a significant role in today’s childhood obesity epidemic. This study’s objective was to assess the prevalence (exposure) and context (power) of the F&B cues in influencer content across three platforms: TikTok, YouTube, and Instagram. The selected influencers were popular with adolescents, with a combined total of more than 34 million followers/subscribers. We employed the YouTube Influencer Marketing Protocol from the World Health Organization (WHO) as our basis for coding. We analysed a total of 360 videos/posts and, of these, 24% contained F&B cues, which is equivalent to 18.1 F&B cues/hour. In total, 77% of the cues were not permitted for children’s advertising, according to WHO criteria, and this was stable across all platforms, with chocolate and sugary confectionery (23%) as the most frequently featured products. Not-permitted F&B had a four-times higher chance of being branded, a five-times higher chance of being described positively, and received significantly more ‘likes’. In 62% of the analysed presentations, the branded product was mentioned, yet only 6% of the content was labelled as advertising. The present analysis delivers further grounds for discussion for policies and regulations of influencer marketing.

## 1. Introduction

Food hauls, taste tests, posts from the latest lunch order or coffee break, and mukbangs (so called “eating broadcast”, which typically showcase overeating on large quantities of food [1]): social media is full of food presentations. Media presentation of food and beverages is an integral part of our lives, and the types of foods we are exposed to shape our preferences and eating behaviours. In fact, there is ample evidence that food advertising and representations are associated with specific brands or food choices (see [2,3,4,5,6,7] for recent reviews and meta analyses). This is even more true when viewers are children, and the food depicted is high in fat, salt, and sugar (HFSS), even more so when viewers are already prone to unhealthy eating habits [8].

It, thus, is crucial to understand how the (digital) food environment created by media for children and adolescents looks. Existing content analyses have focused on traditional TV commercials [9], product placements in TV series and movies [10,11], presentations on websites [12], and in games [13], as well as on social media [14,15,16,17,18,19,20,21,22,23,24]. The collected results all point to a so-called distorted food pyramid, as the majority of food presentations show HFSS foods and beverages low in nutritional value, while, particularly, vegetables and fruits are highly underrepresented [9]. Furthermore, HFSS foods are often depicted in the foreground and are evaluated positively [25]. This has led to some regulatory changes that particularly concern the promotion of HFSS food in commercials targeted at children [26]. 

Yet, social media depictions and promotions of food are still a rather unregulated space [27,28]. At the same time, we lack a comprehensive overview from different countries and among different platforms [29]. However, this is necessary, in order to understand the food environment created online and gain a better grasp on what regulations might be needed. In particular, it is relevant to understand the depictions of food presentation on profiles of social media influencers (SMIs). SMIs are defined as social media users who have gathered a large number of followers and who typically create content around a certain topic, such as sports, travelling, beauty, lifestyle, etc. [30]. They often integrate brand depictions in their content, typically in a very natural, unobtrusive, and authentic way. If they receive compensation from a sponsor, that content needs to be disclosed as such [31]. As consumer protection laws state, it is one of the consumers’ rights to know when they are being exposed to advertising [32]. Yet, not in all cases do brand presentations have a promotional background, as some of the showcased products might be bought by the influencers themselves. Since followers typically build a strong bond with influencers, brand integrations in this manner are generally considered as highly effective at eliciting specific brand and product choices [31]. In fact, several recent experimental studies indicate that young social media users are very likely to follow an influencers’ recommendations of foods, particularly if they are low in nutritional value [25,29,33]. This is because children and adolescents are still developing their knowledge about and handling of persuasive strategies [34]. In addition, adolescents might be particularly open to purchasing suggestions by influencers, as they are vulnerable to peer pressure and might form a particularly strong bond to their online role models [31]. Thus, influencer marketing potentially increases the intake of products presented in this manner, the promotional background notwithstanding. 

With this study, we contribute to the existing body of research on children’s and adolescents’ digital food environment, by examining the presentation of food and beverage brands in SMI content across three social media platforms. With our analysis we build on the *YouTube Social Media Influencer Marketing Protocol* from the World Health Organization (WHO) Regional Office for Europe [35,36], which potentially allows for a comparative analysis with studies that employed or will employ this protocol [37]. We, furthermore, extend the existing body by examining the German-speaking region and by giving an overview of three different platforms. Specifically, we are interested in (A) the exposure (i.e., prevalence of food and beverage types; research question RQ1) and (B) the power (i.e., the persuasive strategy connected to the food and beverage presentation; RQ2) in social media influencer content across three different platforms.

## 2. Materials and Methods

### 2.1. Sample

We selected the most followed influencers on three popular social media platforms, YouTube, TikTok, and Instagram, by consulting three lists on socialblade.com and noxinfluencer.com: (1) top 100 Youtubers sorted by subscribers [38,39,40,41,42,43], (2) top 100 most followed TikTok accounts sorted by followers count [44,45,46], and (3) top 100 most followed Instagram Creator accounts sorted by followers count [47,48,49] in the German-speaking countries of Austria, Germany, and Switzerland. Furthermore, the influencers were selected based on the following criteria: (1) having more than 100,000 subscribers or followers on all three platforms, (2) German content, and (3) popularity among adolescents at the age of 13 to 17 years, with high engagement that was based on the so-called engagement rate. This is determined through the amount of interaction (likes, comments, and shares) the social media content receives in relation to the total number of followers/subscribers [50].

To balance by gender, half of the selected influencers identified themselves as female and half as male, and all influencers can be considered as healthy or normal weight. None of the six influencers was known for their food expertise, but all influencers had previously promoted food products on social media. 

No human participants were involved in this study, so ethical approval was not required. Posts on the platforms were publicly available.

### 2.2. Collecting Social Media Videos and Posts

Data were collected from August 2021 to October 2021. For the content analysis, the last 20 videos and posts of six German-speaking influencers, which were uploaded on YouTube, TikTok, and Instagram before 1 June 2021, were used. Stories on the platforms were excluded because they disappear after 24 h and were no longer visible during data collection. This resulted in a sample of 264 videos or reels and 96 posts (20 per influencer and platform), posted from November 2019 to May 2021. The number of likes per video or post was recorded.

### 2.3. Disclosure of Sponsored Social Media Videos and Posts

The food or beverage was signposted as advertisement in the video itself or in the video/post description, indicated with terms such as “#advert”, “#ad” (German “#Werbung”, “#Anzeige”), “product placement of *company name*”, “competition with *company name*”, or affiliation links. Food, beverages, or brand logos were also obtained in the video description. 

### 2.4. Identifying Foods and Beverages Cue

To explore the prevalence and context of food and beverages cues featured in YouTube, TikTok, and Instagram videos and posts on influencer channels, the *YouTube Social Media Influencer Marketing Protocol (V2 2020)* from the World Health Organization (WHO) Regional Office for Europe [35,36] was used. This WHO protocol includes templates and step-by-step guidance on systematic variables and methods, to ensure replicable and comparable studies of children’s food marketing exposure [37].

Two different kinds of variables were assessed using this protocol: the exposure and the power variables. The exposure variables seized the influencer, the gender identification of the influencer, the number of subscribers, the video link, the date the video was uploaded, the duration of the video, the date the video was coded, the start and end time, the brand name of the food, and the description of the food. Moreover, the power variables captured the cue brand, the context, the description, the presentation, and the reason why the cue was shown [36]. 

All used variables and their coding are presented in the Supplementary Materials (Table S1). 

### 2.5. Obtaining Nutrition Information 

Each food and beverage cue was matched to an entry in the Food Composition Database, called the German Nutrient Database (German: “Bundeslebensmittelschlüssel”; BLS, version 3.02, https://www.blsdb.de/ (accessed on 1 September 2021)). The BLS is a food composition table and contains standard nutritional values per 100 g. For each food and beverage, we obtained energy, total fat, saturated fat, total sugars, added sugars (absent or present), non-sugar sweeteners (absent or present), salt, and fibre from the closest-matching BLS entry, which was required for nutrient profiling. 

### 2.6. Nutrient Profiling

To evaluate nutritional quality, we used the WHO Regional Office for Europe Nutrient Profile Model (NPM), which is an established evidence-based tool [51] and is also included in the *WHO YouTube Social Media Influencer Marketing Protocol* [36]. Based on the WHO NPM, food and beverage cues were assigned to the specific food category. After identifying the appropriate food category, the nutritional content of the food or beverage was evaluated against the thresholds of nutritional composition (total fat, total sugars, added sugar, non-sugar sweeteners, energy, saturated fat, and salt) per 100 g. Afterwards, the products were categorised into three groups; (1) marketing not permitted to children; (2) marketing permitted; and (3) miscellaneous (food or beverage could not be determined due to a lack of relevant nutritional information, e.g., product not identifiable from the video, or it did not fit into any of the food categories). Furthermore, we used the Austrian National Nutrition Commission (NEK) NPM [52], which is based on the WHO NPM, with the same approach but adapted for Austria. Only three products were categorised differently (chicken nuggets, curd, celery juice). Therefore, we focus on the WHO NPM in this content analysis, to ensure replicability and comparability.

### 2.7. Monitoring Coder Reliability

One researcher examined all videos or posts, and another researcher examined a subset of videos or posts (10%, 5.1 h) to assess interrater coding reliability. Cohen’s kappa analysis was calculated to determine consistency among researchers. A Cohen’s kappa agreement of κ > 0.60 was considered acceptable. The interrater reliability for the raters was found to be κ = 0.98 for food cue type (κ range, 0.98–0.99; *p* < 0.001), and κ = 0.68 for WHO NPM groups (κ range, 0.61–0.75; *p* < 0.001).

### 2.8. Statistical Analysis

All variables were analysed descriptively, with frequencies and percentages shown for categorical data. We conducted chi-squared tests to identify potential differences in food and beverage cue categories. To calculate effect sizes, Cramer’s V [53] was used with 0.04 indicating a small, 0.13 a medium, and 0.22 a large effect [54]. 

To assess the association between food and beverage cues, which were not permitted for marketing according to the WHO NPM, and cue brand status, cue description, cue presentation, and likes, a multivariable logistic regression analysis was calculated. In a further step, these regression analyses were adjusted for platform, number of subscribers, and engagement rate. In addition, the number of likes had a skewed distribution and was log-transformed to achieve normal distribution.

Statistical significance was set at *p* < 0.05, and the exact values to *p* < 0.001 were reported.

## 3. Results

### 3.1. Influencer Characteristics

Four influencers were identified in the “Entertainment” channel category, one in the “How-to and Style” category, and one in the “People and Blogs” category. Three influencers (50%) were female, and three (50%) were male, with a median age of 25 years (range, 18–32 years). Five influencers were located in Germany and one in Austria. 

At the time of data collection, the six influencers had approximately a total of 11.4 million followers or subscribers on YouTube (median, 2.2 million (range, 0.2–4.2 million) per influencer), 12.9 million on TikTok (median, 1.3 million (range, 0.2–6.2 million) per influencer) and 10.8 million on Instagram (median, 1.6 million (range, 1.1–3.2 million) per influencer). 

The influencers had uploaded a total of 2079 videos or posts on YouTube (median, 330 (range, 81–706) per influencer), 8285 on TikTok (median, 393 (range, 27–4250) per influencer), and 6320 on Instagram (median, 712 (range, 264–2300) per influencer). 

The median engagement rate (views, likes, comments per post) per influencer was 6.5% (95–CI, 6.0–19.7%) on YouTube, 4.8% (95–CI, 1.5–9.2%) on TikTok, and 1.6% (95–CI, 0.9–2.3%) on Instagram. There was a significant difference between the engagement rates on YouTube and Instagram (*p* = 0.006) and a significant trend between TikTok and Instagram (*p* = 0.054), with a higher one on YouTube and TikTok. The median engagement of likes on YouTube was 9127 (95–CI, 8897–30,120 likes), 68,656 (95–CI, 36,520–252,672 likes) on TikTok, and 28,646 (95–CI, 12,204–39,636 likes) on Instagram. There was a significant difference between the engagement of likes on YouTube and TikTok (*p* = 0.014) and a significant trend between TikTok and Instagram (*p* = 0.054), with a higher one on TikTok and Instagram.

### 3.2. Recording Statistics

A total of 360 videos (120 YouTube videos, 120 TikTok videos, 24 Instagram videos/reels, and 96 Instagram posts) were analysed, which equalled 22.6 h of content (female influencers = 12.4 h, male influencers = 10.2 h; *p* = 0.032). Within the sample, 24% of these videos contained food or beverage cues (n = 409; female influencer n = 214; male influencer n = 195; *p* = 0.074), averaging 18.1 cues per hour (female influencer n = 17.2, male influencer n = 19.1). In half (n = 60) of the YouTube videos, 17% (n = 20) of the TikTok videos, and 6% (n = 7) of the Instagram videos/reels/posts, any food or beverage cue was featured. A total of 273 videos (76%) did not feature any food or beverage cues. 

### 3.3. Nutrient Profiling

**Figure 1:**
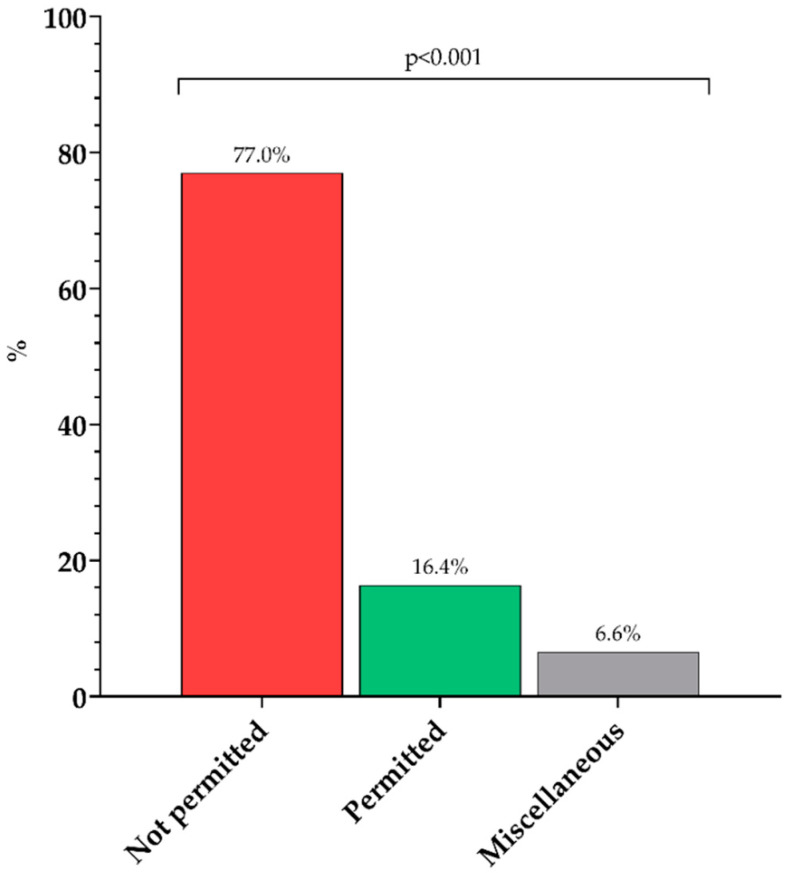
Frequency of food and beverage cues categorised according to the WHO Nutrient Profiling Model. Note: Significant differences were calculated using chi-squared test.

Based on the WHO NPM, there was a significant difference between the categories (χ2(3) = 357.1, *p* < 0.001) and a greater prevalence and rate of food and beverage cues that are not permitted (77.0%) than permitted (16.4%) or miscellaneous (6.6%) (Figure 1). There were no significant differences between the platforms (*p* = 0.148).

Furthermore, food and beverage cues were categorised into 20 different food and beverage types (χ2(20) = 402.31, *p* < 0.001) according to the WHO NPM (Table 1). “Chocolate and sugar confectionery, energy bars, sweet toppings and desserts” was the most frequently featured category, followed by “ready-made and convenience foods and composite dishes”, “beverages—other”, “savoury snacks”, “cakes, sweet biscuits and pastries”, and “sauces, dips and dressings”. “Processed fruit, vegetables and legumes”, and “fresh and frozen fruit, vegetables or legumes” were featured less frequently (Table 1).

### 3.4. Food and Beverage Cues’ Brands, Displays, Description, and Presentation

Table 2a shows the brands, displays, description and presentation of food and beverage cues, split by category according to the WHO Nutrient Profiling Model, while Table 2b is split by the different platforms.

Overall, 68% of food and beverage cues were branded (including food retail establishment brands and supermarket own). Permitted cues for marketing, according to the WHO NPM, were significantly more likely to be unbranded than those that were not permitted. On the contrary, not permitted cues were much more likely to be branded than permitted cues (Table 2a). There was no significant difference between the different platforms (Table 2b).

Concerning the cues’ display, we found no significant difference between the context in which food and beverages were displayed. Overall, the majority of the cues were presented in the context of home (Table 2a) and we observed a significant difference between the platforms (Table 2b).

The cue description in how food and beverage cues were described was significantly different, as not permitted cues were described slightly more positively than permitted ones (Table 2a). Moreover, we could observe a significant difference between the platforms (Table 2b).

Regarding the cues’ presentation, it was more common for cues to be consumed during the video with a verbal reference, compared to those not being consumed. Permitted cues were slightly more frequently consumed with a verbal reference, compared to not permitted cues (Table 2a). There was a significant difference between the platforms (Table 2b).

The reasons why food and beverage cues were featured in the influencers’ videos did not differ significantly within the WHO NPM groups (Table 2a) but did differ within the platforms (Table 2b). Overall, the majority of cues was not explicitly presented as part of a marketing campaign.

The logistic regression analysis revealed that not permitted products had a four-times higher chance of being branded (OR 3.91, 95–CI 1.87–8.17, *p* < 0.001), a five-times higher chance of being described positively (OR 4.61, 95–CI 2.09–10.18, *p* < 0.001), and a four-times higher chance of not being consumed by the influencer themselves (OR 3.88, 95–CI 1.49–10.08, *p* = 0.005), adjusted for the platform, the number of subscribers, and the engagement rate (Cohens d = 0.31). In addition, permitted food and beverage cues were associated with significantly fewer likes (OR 0.42, 95–CI 0.22–0.80, *p* = 0.014) (Table S2). 

### 3.5. Sponsored Social Media Videos/Posts and Disclosure of Adverts

**Figure 2:**
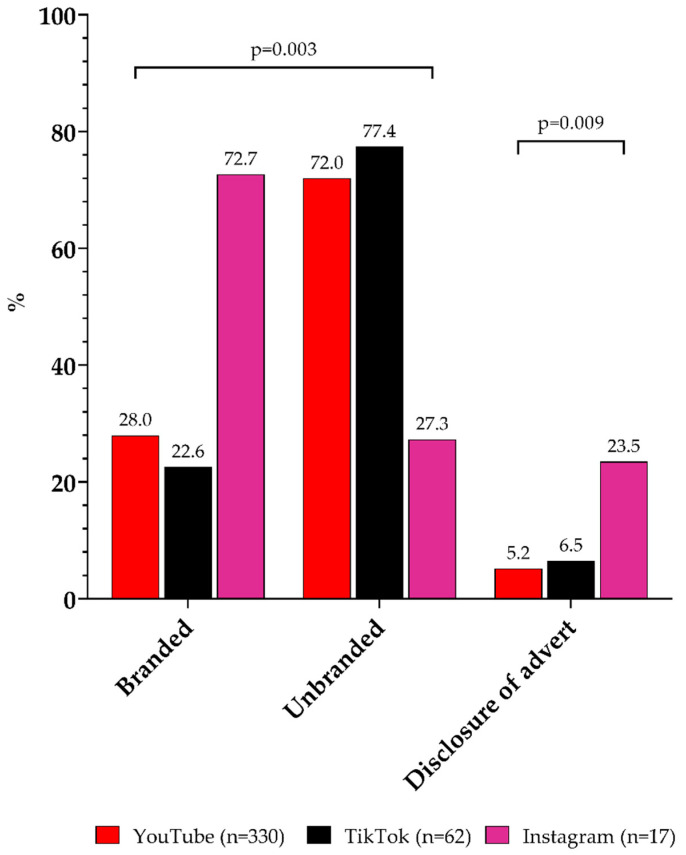
Statement of food and beverage product or brand and disclosure of advert in the video itself and/or video description according to the platforms. Note: Significant differences were calculated using chi-squared test.

In nearly two-thirds (62%) of the food and beverage cues, the product or brand was stated in the video/post description. About one-quarter (29%) of branded food or beverages were stated in the video/post description. Overall, eight different food and beverage companies or brands were named (e.g., Ferrero Int., Mondelez Int., Mars Inc., Coca Cola, McDonald’s, etc.). Regarding the platform comparison, there were significant differences between all categories, with significantly more statements about the product or brand in the description on TikTok (86%, Table S4) compared to YouTube (57%) and Instagram (65%; *p* < 0.001). On YouTube, fewer branded food and beverage products were named compared to unbranded products, approximately the same as on TikTok. In contrast, on Instagram, more branded products were stated in the video description, compared to unbranded products (Figure 2). There were no significant differences between not permitted and permitted cues (Table S3).

Only 6% of the 409 food- or beverage-containing videos or posts were disclosed as advertising in the video itself and/or in the video/post description. There were no significant differences between not permitted and permitted cues (Table S3), but there were significant differences between the platforms (Figure 2, Table S4). Overall, seven disclosures were named (e.g., product placement, affiliation links, advertisement, #ad, etc.).

## 4. Discussion

Influencers are extremely popular communicators, with the specific example of the six influencers we included in this study, who reach approximately 34 million followers or subscribers together on all three platforms. Within the content these influencers create, food plays an important role, with one-quarter of analysed videos and posts on TikTok, YouTube, and Instagram containing food and beverage presentations. Of these presentations, the overwhelming majority was characterised as not permitted food, according to the WHO NPM [36]. This is in line with research from other media outlets and, thus, further adds to the argument of a distorted food pyramid in media targeted at young audiences [10,11,13,25]. The most frequently promoted products in our analysis showed chocolate, convenience food, beverages, and salty snacks. This was stable across all three analysed platforms. With regard to branding, we found that permitted cues for marketing, according to the WHO NPM [36], were significantly more likely to be unbranded than those not permitted. This, together with the result of overwhelming not permitted food presentations, points to the problem that healthy food is lacking a strong brand lobby. While processed products are always associated with a brand, less processed but recommended products such as fruits and vegetables are rarely associated with a brand and, therefore, do not have the funding that ensures prominent exposure.

In addition to being featured more often, not permitted cues were described slightly more positively than permitted ones, especially on TikTok. This adds to the problematic impact of not permitted food presentations, as positive evaluations add to the effectiveness of food presentations [55,56]. However, with regard to consumption, we observed the opposite, with permitted cues being slightly more frequently consumed with a verbal reference compared to not permitted cues. This is a positive sign, as consumption can be characterised as an indicator for prominence [10], which could potentially increase children’s awareness for permitted and thus healthy food choices. As healthy food might need stronger persuasive cues connected to its presentations to truly increase the attractiveness for children, compared to more unhealthy options [57], this can be considered as a relevant step. Future studies should include qualitative approaches, to consider a more in-depth assessment of persuasive cues used in food presentations online. In addition, more effect research with young consumers [25] is needed. 

One option that might also help children to assess food presentations more critically is the use of adequate advertising disclosures [58]. Such disclosures, however, have not been demonstrated to reduce the effect of exposure to advertising on consumption. When advertising is disclosed as such, followers or subscribers are potentially even more likely to consume the advertised products [33]. Yet, the majority of cues were not explicitly presented as part of a marketing campaign. The most apparent marketing was conducted on Instagram, the least on YouTube, and the most branded products also appeared on Instagram. Not all brand presentations in influencer content automatically need to imply a sponsorship relationship, thus, we cannot automatically conclude that influencers are not adequately using ad disclosures in their content. However, the preference for influencers to advertise unpaid products that are not permitted shifts the weight even further in that direction, contributing to the food environment imbalance. Furthermore, their verbal indications of having bought these products themselves might be an even stronger testament to their liking of these foods, which might be particularly persuasive [31]. In this study, we only focused on the content and, thus, only had a limited perspective on the actual through process that was involved in creating the finished output. It, thus, would be highly beneficial to include the perspective of influencers themselves [59]. Future research should consider quantitative and qualitative research with influencers, to examine their content-creation process, ideally with a sample of influencers from different countries, different target groups, and topical backgrounds.

In our study, we were able to shed further light on the presentation of food in online content. Moreover, although we were very systematic in our selection of influencers, there are still limitations to consider. Our data can only speak about the representation in the German-speaking region. However, this could be considered also a strength of the study, as country-specific and country-relevant findings are essential for regulatory discussions. Still, research on other regions and countries, particularly outside the Central European or North American perspective is urgently needed. In addition, as pointed out above, different methodological approaches and more in-depth considerations of the content itself would be a relevant addition to the current literature. By using the WHO NPM for our coding [36], however, international comparability is potentially conceivable. In addition, we used metrics of reach and engagement to select the influencers. By focusing on German-speaking creators, however, we cannot say anything about whether influencers from other countries might not be even more or at least similarly influential in German-speaking countries. In our study, we considered the most relevant social media platforms. Yet, especially in the gaming sector, for example, on Twitch, tendencies to promote not permitted foods can also be identified [60]. It would, therefore, be relevant to consider these platforms as well to get an even more comprehensive view. Since our analysis was a content analysis, it is impossible to make any statements about possible effects. However, the current empirical evidence tells a relatively clear story regarding the dominance of nutritionally poor foods in the digital food environment of children and their food preferences [5,6,7]. As pointed out by the Ending Childhood Obesity report in 2016 [61], marketing of unhealthy foods and sugar-sweetened beverages has a clear link to childhood obesity, according to the unequivocal evidence [62,63]. The exposure to the marketing of unhealthy foods continues to be a significant problem, which calls for reform to protect all children equally, despite the growing number of industry-led voluntary measures. Therefore, reducing children’s exposure to marketing and its influence should be a part of any attempt to tackle childhood obesity. Thus, calls for actions by regulators and content creators to rethink and minimise the dominance of not permitted foods online is also supported by our results.

## 5. Conclusions

The high relevance of foods considered as inappropriate for marketing targeted children and, thus, not permitted [36] in influencer content makes it clear that there is an urgent need for regulations that also include social media more rigorously [27]. Effect studies already show that what influencers on social media eat and recommend is considered very attractive to their young audiences [29,33,64]. Therefore, the food environment they create with their content is crucial. As the vast majority of influencer marketing is for nutritionally poor food and beverages, policymakers and social media platforms need to consider this fact in their regulatory decisions. This study further highlights the prevalence of particularly nutritionally poor food in content targeted at children, which might further tip the scale in favour of such food. Due to the popularity of platforms such as TikTok, YouTube, and Instagram among children, there is a need to develop effective policies and to ensure policy adherence. Ideally, an international approach and a feasible and scalable system to monitor the extent of inappropriate marketing needs to be developed.

## Figures and Tables

**Table 1:** Food and beverage types based on the WHO Nutrient Profiling Model ordered by frequency of appearance in influencer videos (n = 264) or posts (n = 96).

Food & Beverage Type Based on WHO Nutrient Profiling Model	Frequency (n = 403)	%	*p* Value ^1^
Chocolate and sugar confectionery, energy bars, sweet toppings and desserts	92	22.8%	**<0.001**
Ready-made and convenience foods and composite dishes	39	9.7%	
Beverages—Other	38	9.4%	
Savoury snacks	34	8.4%	
Cakes, sweet biscuits and pastries; other sweet bakery wares and dry mixes for making such	30	7.4%	
Sauces, dips and dressings	28	6.9%	
Processed fruit, vegetables and legumes	22	5.5%	
Fresh and frozen fruit, vegetables or legumes	17	4.2%	
Fresh or dried pasta, rice and grains	16	4.0%	
Beverages—Milk drinks	14	3.5%	
Cheese	14	3.5%	
Bread, bread products and crisp breads	13	3.2%	
Yoghurts, sour milk, cream and other similar foods	12	3.0%	
Beverages—Juices	10	2.5%	
Processed meat, poultry, fish and similar	7	1.7%	
Edible ices	6	1.5%	
Breakfast cereals	5	1.2%	
Butter and other fats and oils	3	0.7%	
Fresh and frozen meat, poultry, fish and similar	2	0.5%	
Beverages—Energy drinks	1	0.2%	

^1^ Significant differences were calculated using chi-squared test; Significant findings are in bold.

**Table 2:** (**a**). Food and beverage cues’ brands, displays, description, and presentation, split by category according to the WHO Nutrient Profiling Model. (**b**). Food and beverage cues’ brands, displays, description, and presentation, split by category between the platforms.

**(a)**						
	**Overall** **(n = 409)**	**Not Permitted** **(n = 315)**	**Permitted** **(n = 67)**	**Miscellaneous** **(n = 27)**	***p* Value ^1^**	**Cramers’ V**
	**n (%)**	**n (%)**	**n (%)**	**n (%)**		
*Brand*						
Branded	224 (54.8)	190 (60.3)	28 (41.8)	6 (22.2)	**<0.001**	0.19
Food retail establishment	23 (5.6)	18 (5.7)	2 (3.0)	3 (11.1)		
Supermarket own	29 (7.1)	23 (7.3)	6 (9.0)	0 (0.0)		
Unbranded	133 (32.5)	84 (26.7)	31 (46.3)	18 (66.7)		
*Cue display*						
Eating-out meal	40 (9.8)	29 (9.2)	7 (10.4)	4 (14.8)	0.699	0.07
Supermarket	9 (2.2)	9 (2.9)	0 (0.0)	0 (0.0)		
Home	335 (81.9)	257 (81.6)	56 (83.6)	22 (81.5)		
Other	25 (6.1)	20 (6.3)	4 (6.0)	1 (3.7)		
*Cue description*						
Positive	215 (52.6)	166 (52.7)	29 (43.3)	20 (74.1)	**0.009**	0.13
Negative	21 (5.1)	12 (3.8)	8 (11.9)	1 (3.7)		
Neutral	173 (42.3)	137 (43.5)	30 (44.8)	6 (22.2)		
*Cue presentation*						
Consumed and verbal reference	260 (63.6)	189 (60.0)	48 (71.6)	23 (85.2)	**0.009**	0.14
Consumed, no verbal reference	35 (8.6)	25 (7.9)	9 (13.4)	1 (3.7)		
Not consumed and verbal reference	58 (14.2)	49 (15.6)	8 (11.9)	1 (3.7)		
Not consumed, no verbal reference	56 (13.7)	52 (16.5)	2 (3.0)	2 (7.4)		
*Reason cue was featured*						
Not explicit marketing (campaign)	394 (96.3)	300 (95.2)	67 (100.0)	27 (100.0)	0.326	0.08
Gifted by brand	5 (1.2)	5 (1.6)	0 (0.0)	0 (0.0)		
Paid by brand	10 (2.4)	10 (3.2)	0 (0.0)	0 (0.0)		
**(b)**						
	**Overall** **(n = 409)**	**YouTube** **(n = 330)**	**TikTok** **(n = 62)**	**Instagram** **(n = 17)**	***p* Value ^1^**	**Cramers’ V**
	**n (%)**	**n (%)**	**n (%)**	**n (%)**		
*Brand*						
Branded	224 (54.8)	186 (56.4)	31 (50)	7 (41.2)	0.167	0.11
Food retail establishment	23 (5.6)	22 (6.7)	1 (1.6)	0 (0)		
Supermarket own	29 (7.1)	21 (6.4)	7 (11.3)	1 (5.9)		
Unbranded	133 (32.5)	101 (30.6)	23 (37.1)	9 (52.9)		
*Cue display*						
Eating-out meal	40 (9.8)	34 (10.3)	6 (9.7)	0 (0)	**<0.001**	0.31
Supermarket	9 (2.2)	9 (2.7)	0 (0)	0 (0)		
Home	335 (81.9)	279 (84.5)	48 (77.4)	8 (47.1)		
Other	25 (6.1)	8 (2.4)	8 (12.9)	9 (52.9)		
*Cue description*						
Positive	215 (52.6)	159 (48.2)	45 (72.6)	11 (64.7)	**0.003**	0.14
Negative	21 (5.1)	17 (5.2)	4 (6.5)	0 (0)		
Neutral	173 (42.3)	154 (46.7)	13 (21)	6 (35.3)		
*Cue presentation*						
Consumed and verbal reference	260 (63.6)	225 (68.2)	25 (40.3)	10 (58.8)	**<0.001**	0.23
Consumed, no verbal reference	35 (8.6)	19 (5.8)	16 (25.8)	0 (0)		
Not consumed and verbal reference	58 (14.2)	42 (12.7)	15 (24.2)	1 (5.9)		
Not consumed, no verbal reference	56 (13.7)	44 (13.3)	6 (9.7)	6 (35.3)		
*Reason cue was featured*						
Not explicit marketing (campaign)	394 (96.3)	329 (99.7)	52 (83.9)	13 (76.5)	**<0.001**	0.30
Gifted by brand	5 (1.2)	0 (0)	5 (8.1)	0 (0)		
Paid by brand	10 (2.4)	1 (0.3)	5 (8.1)	4 (23.5)		

(**a**) ^1^ Significant differences were calculated using chi-squared test; percentages in the overall column refer to the group percentage within each category. Percentages in the “not permitted”, “permitted”, and “miscellaneous” columns refers to the percentage within the group; Significant findings are in bold. (**b**) ^1^ Significant differences were calculated using chi-squared test; percentages in the overall column refer to the group percentage within each category. Percentages in the “YouTube”, “TikTok”, and “Instagram” columns refers to the percentage within the group; Significant findings are in bold.

## Data Availability

The data presented in this study are available on request from the corresponding author.

## Supplementary Materials

The following supporting information can be downloaded at: https://www.mdpi.com/article/10.3390/ijerph191710911/s1. Table S1: All used exposure and power variables and their coding; Table S2: Association between cues which are permitted to marketing according to the WHO Nutrient Profile Model and cue’s brand status, description, presentation and number of likes (n = 315); Table S3: Statement of food & beverage product or brand and disclosure of advert in the video itself and/or video description, split by category according to the WHO Nutrient Profiling Model; Table S4: Statement of food & beverage product or brand and disclosure of advert in the video itself and/or video description, split by the platforms.
