# Supplementary material for: Promotion of Food and Beverages by German-Speaking Influencers Popular with Adolescents on TikTok, YouTube and Instagram
Source: Int J Environ Res Public Health. 2022 Sep 1;19(17):10911. doi: 10.3390/ijerph191710911 (PMC9518047; doi:10.3390/ijerph191710911)
Supplement: Supplementary file 1 [file ijerph-19-10911-s001.zip › ijerph-1835246-supplementary.pdf]

Table S1: All used exposure and power variables and their coding.

Table S2: Association between cues that are permitted for marketing according to the WHO Nutrient Profile Model and cue's brand status, description, presentation, and the number of likes (n=315).

Table S3: Statement of food & beverage product or brand and disclosure of advert in the video itself and/or video description, split by category according to the WHO Nutrient Profiling Model.

Table S4: Statement of food & beverage product or brand and disclosure of advert in the video itself and/or video description, split by the platforms.

Table S1. All used exposure and power variables and their coding.

VARIABLE TYPE	VARIABLE	DESCRIPTION OF DATA REQUIRED
	ID	Each line has a unique identification number
	Post ID	Each video has a unique identification number
	Cue ID	Each cue has a unique identification number
	Social media platform	Social media platforms
		1=YouTube
		2=TikTok
		3=Instagram
**EXPOSURE**	Influencer	Name of influencer
**EXPOSURE**	Influencer sex	Influencer sex identification
		1=Female
		2=Male
**EXPOSURE**	Influencer age	Age of influencer (year)
**EXPOSURE**	Influencer area	1=Austria
		2=Germany
**EXPOSURE**	Language	Language of influencer
		1=German
		2=English
**EXPOSURE**	Categories	Categories of influencer
		1=Entertainment
		2=How to & Style
		3=People & Blogs
**EXPOSURE**	Channel tags	Channel or hashtags of influencer
**EXPOSURE**	Number of subscribers	Number of subscribers to channel or follower stated on influencer profile
**EXPOSURE**	Total videos or posts per platform	Total uploaded videos or posts of influencer
**EXPOSURE**	Engagement rate	Engagement Rate (average interactions per post) in % of influencer
**EXPOSURE**	Engagement likes	Average likes per post (engagement rate) of influencer
**EXPOSURE**	Number of likes	Number of likes per video/post
**EXPOSURE**	Video link	Hyperlink to video
**EXPOSURE**	Date video uploaded	DD/MM/YYYY
**EXPOSURE**	Video or reel	Is this a video/reel or a post?
		0=no
		1=yes
**EXPOSURE**	Duration of video	e.g. 13:50
**EXPOSURE**	Date video coded	DD/MM/YYYY
**EXPOSURE**	Video contains food or beverage	Video contains food or beverage
		0=no
		1=yes
**EXPOSURE**	Start time of cue	Minutes and seconds of video elapsed when cue appears
**EXPOSURE**	End time of cue	Minutes and seconds of video elapsed when cue ends
**EXPOSURE**	2nd cue exposure start time	Minutes and seconds of video elapsed when cue appears again
**EXPOSURE**	2nd cue exposure end time	Minutes and seconds of video elapsed when cue ends
**EXPOSURE**	3rd cue exposure start time	Minutes and seconds of video elapsed when cue appears again
**EXPOSURE**	3rd cue exposure end time	Minutes and seconds of video elapsed when cue ends
**EXPOSURE**	4th cue exposure start time	Minutes and seconds of video elapsed when cue appears again
**EXPOSURE**	4th cue exposure end time	Minutes and seconds of video elapsed when cue ends
**EXPOSURE**	Cue exposed constantly	0=no

		1=yes
**EXPOSURE**	Total cue duration	Cumulative duration (all exposure durations recorded for this cue combined)
**EXPOSURE**	Food product brand name	Name and (if present) brand name of the product (e.g. "McDonald's Big Mac" or "spaghetti bolognese")
**EXPOSURE**	Detailed description of food product	The description of the product should be thorough. The product needs to be identifiable for the purposes of collecting nutritional information later. Include flavour or brand variant (e.g. 'chocolate coated, cream-filled biscuit' rather than just 'biscuit'). If coding a fast food advertisement, note down all items in a meal bundle (e.g. if McDonald's Happy Meal record that it comprises of e.g. a hamburger, a bottle of water and a fruit bag here).
**EXPOSURE**	WHO Regional Office for Europe nutrient profile model (NPM) food category code	1=Chocolate and sugar confectionery, energy bars, sweet toppings and desserts (Includes chocolate and other products containing cocoa; white chocolate; jelly, sweets, boiled sweets; chewing gum and bubble gum; caramels; liquorice sweets; spreadable chocolate and other sweet sandwich toppings; nut spreads including peanut butter; cereal, granola and muesli bars; marzipan. Does not include chocolate-flavoured breakfast cereals; cakes and pastries; biscuits and other baked goods covered in chocolate). Not permitted to be marketed to children.
		2=Cakes, sweet biscuits and pastries; other sweet bakery wares, and dry mixes for making such (Includes pastries; croissants; cookies/biscuits; sponge cakes; wafers; fruit pies; sweet buns; chocolate-covered biscuits; cake mixes and batters. Do not include bread and bread products). Not permitted to be marketed to children.
		3=Savoury snacks (Includes popcorn and maize corn; seeds; nuts and mixed nuts; savoury biscuits and pretzels; other snacks made from rice, maize, dough or potato). Marketing to children not permitted if added sugars exceed 0g/100g or if salt exceeds 0.1g per 100g.
		4a=Beverages - Juices (Includes 100% fruit and vegetable juices; juices reconstituted from concentrate, and smoothies). Not permitted to be marketed to children.
		4b=Beverages - Milk drinks (Includes milks and sweetened milks; almond, soya, rice and oat milks. Does not include cream). Marketing to children not permitted if total fat exceeds 2.5g/100g or if added sugars/non-sugar sweeteners exceed 0g/100g.
		4c=Beverages - Energy drinks (There is no agreement on a definition of energy drinks. However, such a category of drinks includes a variety of non-alcoholic beverages. While caffeine is considered the main ingredient, a number of other substances are often present. The most common of these include guarana, taurine, glucuronolactone and vitamins. A common feature is that these beverages are marketed for their actual or perceived effects as stimulants, energizers and performance enhancers). Not permitted to be marketed to children.
		4d=Beverages - Other (Including cola, lemonade, orangeade; sweetened beverages, mineral and/or flavoured waters (including aerated) with added sugars or sweetener. Does not include 100% fruit and vegetable juices; milk drinks). Marketing to children not permitted if added sugars/non-sugar sweeteners exceed 0g/100g.

		5=Edible ices (Including ice cream, frozen yoghurt, iced lollies and sorbets). Not permitted to be marketed to children.
		6=Breakfast cereals (Including oatmeal; cornflakes; chocolate breakfast cereals; mueslis). Marketing to children not permitted if total fat exceeds 10g/100g, or if total sugars exceed 15g/100g or if salt exceeds 1.6g/100g.
		7=Yoghurts, sour milk, cream and other similar foods (Including yoghurt; kephir; buttermilk; flavoured sour; fermented milk and drinking yoghurt; fromage frais; cheese-based and other yoghurt substitutes; yoghurt products containing additional ingredients (such as fruit; muesli); cream. Does not include milks and sweetened milks; almond, rice and oat milks). Marketing to children not permitted if total fat exceeds 2.5g/100g, or if saturated fat exceeds 2.0g/100g, or if total sugars exceed 10g/100g or if salt exceeds 0.2g/100g.
		8=Cheese (Including medium-hard and hard cheeses; soft cheeses; fresh cheese (such as ricotta, mozzarella); grated or powdered cheese; cottage cheese; processed cheese spreads). Marketing to children not permitted if total fat exceeds 20g/100g, or if salt exceeds 1.3g/100g.
		9=Ready-made and convenience foods and composite dishes (Including pizzas; lasagne and other pasta dishes with sauces; quiches; ready meals; ready-made sandwiches; filled pastas; soups and stews (packaged or tinned); mixes and dough). Marketing to children not permitted if total fat exceeds 10g/100g, or if saturated fat exceeds 4g/100g, or if total sugars exceed 10g/100g or if salt exceeds 1g/100g or if energy exceeds 225kcal/100g.
		10=Butter and other fats and oils (Including butter; vegetable oils; margarines and spreads). Marketing to children not permitted if saturated fat exceeds 20g/100g, or if salt exceeds 1.3g/100g.
		11=Bread, bread products and crisp breads (Includes ordinary bread (containing cereal, leavens and salt); gluten-free bread; unleavened bread; crisp breads; rusks and toasted breads. Does not include sweet biscuits; pastries; cakes). Marketing to children not permitted if total fat exceeds 10g/100g, or if total sugars exceed 10g/100g, or if salt exceeds 1.2g/100g.
		12=Fresh or dried pasta, rice and grains (Does not include filled pasta and pasta in sauce). Marketing to children not permitted if total fat exceeds 10g/100g, or if total sugars exceed 10g/100g, or if salt exceeds 1.2g/100g.
		13=Fresh and frozen meat, poultry, fish and similar (Including eggs). Permitted to be marketed to children.
		14=Processed meat, poultry, fish and similar (Including sausage, ham, bacon; chicken nuggets; smoked and pickled fish; tinned fish in brine or oils; fish fingers and breaded/battered fish. Does not include pepperoni pizza). Marketing to children not permitted if total fat exceeds 20g/100g, or if salt exceeds 1.7g/100g.
		15= Fresh and frozen fruit, vegetables or legumes (Including fruit and vegetables; legumes; starchy vegetables, roots and tubers. Does not include tinned fruits, vegetables and legumes; fruit in syrup; dried fruit; frozen fruit with added sugar). Permitted to be marketed to children.
		16=Processed fruit, vegetables and legumes (Including tinned fruit, vegetables and legumes; dried fruit; dried vegetables

		and legumes; marmalade; jams; pickled vegetables and fruit; stewed fruits; fruit peel; frozen French fries' frozen fruit with added sugar. Does not include fruit juice). Marketing to children not permitted if total fat exceeds 5g/100g, or if total sugars exceed 10g/100g, or if added sugars exceed 0g/100g, or if salt exceeds 1g/100g.
		17=Sauces, dips and dressings (Including salad dressings; tomato ketchup; mayonnaise; ready-to-use dips; soya sauce; mustard and mustard flour). Marketing to children not permitted if total fat exceeds 10g/100g, or if added sugars exceeds 0g/100g or if salt exceeds 1g/100g.
**EXPOSURE**	Total fat of the product	Grams per 100g/ml product
**EXPOSURE**	Saturated fat of the product	Grams per 100g/ml product
**EXPOSURE**	Total sugars of the product	Grams per 100g/ml product
**EXPOSURE**	Added sugars of the product	0=absent
		1=present
**EXPOSURE**	Non-sugar sweeteners of the product	0=absent
		1=present
**EXPOSURE**	Salt of the product	Grams per 100g/ml product
**EXPOSURE**	Energy of the product	Kcal per 100g/ml product
**EXPOSURE**	Fibre of the product	Grams per 100g/ml product
**EXPOSURE**	Marketing permitted according to WHO NPM	0=no
		1=yes
		2=could not be determined due to a lack of relevant nutritional information
**EXPOSURE**	National Nutrition Commission (NEK) nutrient profile model (NPM) food category code	1=Chocolate and sugar confectionery, energy bars, sweet toppings and desserts
		2=Cakes, sweet biscuits and pastries; other sweet bakery wares, and dry mixes for making such
		3=Edible ices
		4=Savoury snacks
		5a=Beverages - Water
		5b=Beverages - Juices
		5c=Beverages - Energy drinks
		5d=Beverages - Other
		6=Breakfast cereals
		7=Bread, bread products and crisp breads
		8=Plant-based drinks, plant-based desserts, similar foods
		9a=Consumer milk, mixed milk products made from non-fermented milk
		9b=Yoghurts, sour milk, cream and other similar foods
		9c=Aged cheese (hard, semi-hard, soft, sour milk cheese), firm mozzarella cheese
		9d=Cream cheese and other cheeses
		10=Savoury spreads
		11=Nuts and seeds
		12=Butter and other fats and oils
		13=Ready-made and convenience foods and composite dishes
		14=Fresh or dried pasta, rice and grains
		15=Fresh and frozen meat, poultry, fish and similar
		16a=Processed meat, sausages, ham, bacon and similar
		16b=Processed fish, crayfish, molluscs (neither breaded nor deep-fried)

		16c=Other processed meat, poultry and similar (neither breaded nor deep-fried)
		16d=Fried, breaded and pre-fried food
		17=Fresh and frozen fruit, vegetables or legumes
		18=Processed fruit, vegetables and legumes (neither breaded nor deep-fried)
		19=Sauces, dips and dressings
**EXPOSURE**	Marketing permitted according to NEK NPM	0=no
		1=yes
		2=could not be determined due to a lack of relevant nutritional information
**POWER**	Cue brand status	1=branded
		2=Food retail establishment (FRE)
		3=Supermarket own
		4=Unbranded
		5=Miscellaneous
**POWER**	Cue context	1=Eating-out meal
		2=Supermarket
		3=Home
		4=Other
**POWER**	Cue description	1=Positive
		2=Negative
		3=Neutral
**POWER**	Cue presentation	1=Consumed and verbally referenced
		2=Consumed and not verbally referenced
		3=Not consumed and verbally referenced
		4=Not consumed and not verbally referenced
**POWER**	Why cue featured	1=non-explicit marketing
		2=gifted endorsement
		3=paid endorsement
**POWER**	Notes	Any other information that may be relevant to record
**STATEMENT**	Text in description	Description of the video/post
**STATEMENT**	Food in description	Food or beverage product or brand stated in video/post description
		0=no
		1=yes
**STATEMENT**	Shown in description	Branded or unbranded food or beverage stated in video/post description
		1=food or beverage brand
		2=nonfood brand
		3=unbranded food or beverage
		4=influencer but no food or beverage
**STATEMENT**	Brand in description	Featured brand in video description
**DISCLOSURE**	Marked as advert in description	Marked as advertising in video/post description
		0=no
		1=yes
**DISCLOSURE**	How advert marked	How advert was marked
**DISCLOSURE**	Marked as advert in video itself	Marked as advertising in video itself
		0=no
		1=yes

Note: The exposure and the power variables were assessed using the YouTube social media influencer marketing protocol (V2 2020) from the World Health Organization (WHO) Regional Office for Europe (21,22).

Table S2. Association between cues which are permitted to marketing according to the WHO Nutrient Profile Model and cue's brand status, description, presentation and number of likes (n=315).

	Marketing not permitted according to the WHO Nutrient Profile Model									
	**Crude model** *(Cohens d = 0.19)*				**Adjusted for platform, and number of subscribers** *(Cohens d = 0.25)*			**Adjusted for platform, number of subscribers, and engagement rate** *(Cohens d = 0.31)*		
	n (%) / mean (SD)	*OR*	*95%CI*	*p-value*[1]	*OR*	*95%CI*	*p-value*[1]	*OR*	*95%CI*	*p-value*[1]
**Cue brand status**										
Unbranded	231 (86.5)		1							
Branded	84 (73.0)	3.69	1.97-6.94	**<0.001**	3.84	1.99-7.39	**<0.001**	3.91	1.87-8.17	**<0.001**
**Cue description**										
Negative/Neutral	166 (85.1)		1							
Positive	149 (79.7)	2.96	1.55-5.65	**<0.001**	3.52	1.73-7.17	**<0.001**	4.61	2.09-10.18	**<0.001**
**Cue presentation (consumption)**										
Consumed	214 (79.0)		1							
Not Consumed	101 (91.0)	3.24	1.43-7.34	**0.005**	3.39	1.47-7.86	**0.004**	3.88	1.49-10.08	**0.005**
**Cue presentation (verbally referenced)**										
Verbally referenced	238 (81.0)		1							
Not verbally referenced	77 (87.5)	1.50	0.68-3.28	0.312	1.38	0.60-318	0.453	1.54	0.61-3.89	0.363
**Number of likes**[2]	4.26 (0.52)	0.42	0.22-0.80	**0.014**	0.50	0.24-1.07	0.075	0.32	0.10-1.05	0.060

[1] Results are based on multivariable logistic regression analysis. [2] Number of likes had skewed distribution and were log trans-formed to achieve normal distribution. Data are presented as odds ratios (OR) with 95% confidence interval (95%CI).

Table S3. Statement of food & beverage product or brand and disclosure of advert in the video itself and/or video description, split by category according to the WHO Nutrient Profiling Model.

	**Overall (n = 409)** *n (%)*	**Not permitted (n = 315)** *n (%)*	**Permitted (n = 67)** *n (%)*	**Miscellaneous (n = 27)** *n (%)*	***p value*[1]**	***Cramers' V***
Product or brand stated in video description	253 (61.9)	193 (61.3)	41 (61.2)	19 (70.4)	0.641	0.047
Branded food or beverage	73 (28.9)	62 (32.1)	8 (19.5)	3 (15.8)	0.115	0.131
Unbranded food or beverage	180 (71.1)	131 (67.9)	33 (80.5)	16 (84.2)		
Disclosed as advertising in video itself and/or video/post description	25 (6.1)	23 (7.3)	2 (3)	0 (0)	0.159	0.095

[1] Significant differences were calculated using Chi-Square Test; Percentages in the overall column refer to the group% within each category. Percentages in the "not permitted", "permitted", and "miscellaneous" columns refers to the % within the group.

Table S4. Statement of food & beverage product or brand and disclosure of advert in the video itself and/or video description, split by the platforms.

	**Overall (n = 409)** *n (%)*	**YouTube (n = 330)** *n (%)*	**TikTok (n = 62)** *n (%)*	**Instagram (n = 17)** *n (%)*	***p value*[1]**	***Cramers' V***
Product or brand stated in video description	253 (61.9)	189 (57.3)	53 (85.5)	11 (64.7)	**<0.001**	0.208
Branded food or beverage	73 (28.9)	53 (28)	12 (22.6)	8 (72.7)	**0.003**	0.212
Unbranded food or beverage	180 (71.1)	136 (72)	41 (77.4)	3 (27.3)		
Disclosed as advertising in video itself and/or video/post description	25 (6.1)	17 (5.2)	4 (6.5)	4 (23.5)	**0.009**	0.153

[1] Significant differences were calculated using Chi-Square Test; Percentages in the overall column refer to the group% within each category. Percentages in the YouTube", "TikTok", and "Instagram" columns refers to the % within the group.
